# Reduced circulating STOX1 is associated with inflammatory cytokines and insulin resistance in obese individuals: a cross-sectional study

**DOI:** 10.3389/fnut.2026.1734606

**Published:** 2026-01-22

**Authors:** Yanping Wang, Mingliang Xiang, Xianmei Jiang, Mingsha Shuai, Li Jian, Shan Geng

**Affiliations:** 1 Central Laboratory of the People’s Hospital of Dazu, The Affiliated Dazu’s Hospital of Chongqing Medical University, Chongqing, China; 2 Department of Endocrinology, The Affiliated Dazu’s Hospital of Chongqing Medical University, Chongqing, China

**Keywords:** adipokines, inflammation, metabolism, obesity, STOX1

## Abstract

**Introduction:**

Excess adiposity drives adipose tissue dysfunction and metabolic disease, including type 2 diabetes and cardiovascular disorders. Building on our prior GEO-based analyses that nominated Storkhead box 1 (STOX1) as a candidate biomarker of obesity-related adipose inflammation, we compared circulating STOX1 between individuals with normal weight and those with overweight/obesity and examined its metabolic correlates.

**Methods:**

In 476 volunteers, we quantified serum STOX1, adipokines, and clinical parameters; we further contrasted STOX1 and adiponectin between groups and performed an oral glucose tolerance test (OGTT) to assess glycemic effects on STOX1.

**Results:**

Serum STOX1 was significantly lower in overweight/obese (OW/OB) participants than controls (controls: 1.27 ± 1.40 µg/L; OW/OB: 0.69 ± 0.77 µg/L; *p* < 0.001), accompanied by reduced adiponectin in OW/OB. Partial correlations showed inverse associations of STOX1 with metabolic and inflammatory indices and a positive association with adiponectin. In multivariable linear regression, systolic blood pressure (SBP), tumor necrosis factor-alpha (TNF-α), and body fat percentage independently predicted STOX1 (Y_STOX1 = 2.569 – 0.110 × SBP – 0.272 × TNF-α – 0.106 × body fat %; *R* = 0.37, *R*^2^ = 0.13).

**Discussion:**

During OGTT, hyperglycemia suppressed circulating STOX1, suggesting glucose-dependent regulation of its secretion/release. Collectively, these findings indicate that decreased serum STOX1 tracks adverse metabolic and inflammatory profiles in obesity and support its potential utility as a noninvasive biomarker of adipose tissue inflammation in overweight/obese individuals.

## 1 Introduction

Over the past few decades, obesity has progressively emerged as a significant global health challenge. Data from the China Chronic Disease and Risk Factors Surveillance (CCDRFS) program indicate that the prevalence of obesity in adults in China more than doubled between 2004 (3.1%) and 2018 (8.1%), based on the estimated 2018 prevalence, obesity affects approximately 85 million people in China (1). Studies suggest that the global obese population is projected to increase to 1.12 billion by 2030. The rising prevalence of obesity necessitates immediate attention through comprehensive research on behavioral risk factors. Importantly, the rapid increase in obesity incidence is anticipated to substantially elevate the prevalence of hypertension, diabetes, hypercholesterolemia, chronic obstructive pulmonary disease, and cancer, thus posing significant risks to public health (2). However, current prevention and treatment strategies have achieved only limited success, underscoring the need to elucidate the pathophysiological mechanisms of obesity and to identify novel diagnostic markers or therapeutic targets. Studies have shown that adipose tissue plays a central role in the pathogenesis of obesity and its related metabolic disorders (3). Under sustained energy surplus, adipose tissue stores excess energy as triglycerides through adipocyte proliferation and hypertrophy. When lipid storage capacity is exceeded, this leads to adipocyte dysfunction, ectopic lipid deposition, insulin resistance, and chronic inflammation (4). The latest research has indicates the significance of the dynamic changes in adipocytes and their surrounding immune cells, angiogenesis and extracellular matrix remodeling in regulating the scalability and functional integrity of this tissue. In obese tissues, macrophages are recruited and trigger an inflammatory response. The quantity of dead adipocytes is closely related to the pathophysiological outcomes of obesity, including insulin resistance and hepatic steatosis (5). Adipose tissue is a dynamic and active endocrine organ that regulates various physiological and pathological processes, such as hemostasis, blood pressure, lipid and sugar metabolism, inflammation, and atherosclerosis, by secreting multiple adipokines (6). Obesity disturbs this regulatory function, enhancing the release of pro-inflammatory cytokines [e.g., Tumor necrosis factor-alpha (TNF-α), Interleukin-6 (IL-6)] and reducing anti-inflammatory adipokines like adiponectin and leptin (7). This imbalance contributes to a state of chronic low-grade inflammation that drives obesity-related diseases. Therefore, identifying novel anti-inflammatory adipokines may help improve adipose dysfunction and metabolic outcomes in obesity.

In our earlier bioinformatics and machine learning-based analysis, we identified Storkhead box 1 (STOX1) as a metabolism-related gene with diagnostic potential in obesity. Real-time reverse transcriptase-polymerase chain reaction (RT-qPCR) validation in mouse models revealed that STOX1 expression was significantly reduced in white adipose tissue of obese mice (8). Therefore, we are suggesting its involvement in glucose and lipid metabolism. STOX1 is a transcription factor that shares structural and functional similarities with homeobox regulators (9). STOX1 encodes a winged-helix transcription factor that has attracted sustained interest as a regulator of placental development and a candidate susceptibility gene in genetic forms of preeclampsia (10). Mechanistic studies support a model in which STOX1 modulates trophoblast programs required for effective placentation, including EVT-mediated signaling that coordinates endothelial responses, immune cell recruitment, and uterine-vascular remodeling (11). In parallel, STOX1 has been implicated in placental stress-adaptation pathways, with reported roles in controlling oxidative/nitrosative stress balance and mitochondrial homeostasis—processes that align closely with core features of preeclampsia pathophysiology. Notably, alternative STOX1 isoforms (often described as STOX1A and STOX1B) may exert non-equivalent effects, and isoform imbalance has been proposed as a driver of trophoblast dysfunction and PE-like molecular phenotypes (12). Existing research primarily focuses on its roles in cell cycle progression, oxidative stress, placental function, and maternal blood pressure regulation (13–15). Moreover, STOX1 also has significant implications in various diseases, such as fetal development and maternal blood pressure regulation (16), pulmonary artery remodeling, promote mitotic entry and proliferation of inner ear epithelial cells (17). For now, there are few studies on STOX1 in the regulation of metabolic diseases in obese individuals, and the mechanism is still unclear. In additional, cure the lack of clinical population studies, the differences in circulating STOX1 between overweight/obese (OW/OB) and normal individuals, as well as its pathophysiological processes and mechanisms involved in regulating obesity-related metabolic diseases remain unclear. Therefore, this study aims to investigate the differences in circulating STOX1 levels between OW/OB and normal individuals, clarify how it involved in obesity-linked metabolic diseases.

In this study, we examined clinical features and circulating STOX1 and Adiponectin levels in obese patients and normal individuals and analyzed the correlations between circulating STOX1 and other parameters as well as the occurrence of OW/OB. Explored the effects of OGTT on circulating STOX1 levels in humans.

## 2 Materials and methods

### 2.1 Study population

**Figure 1:**
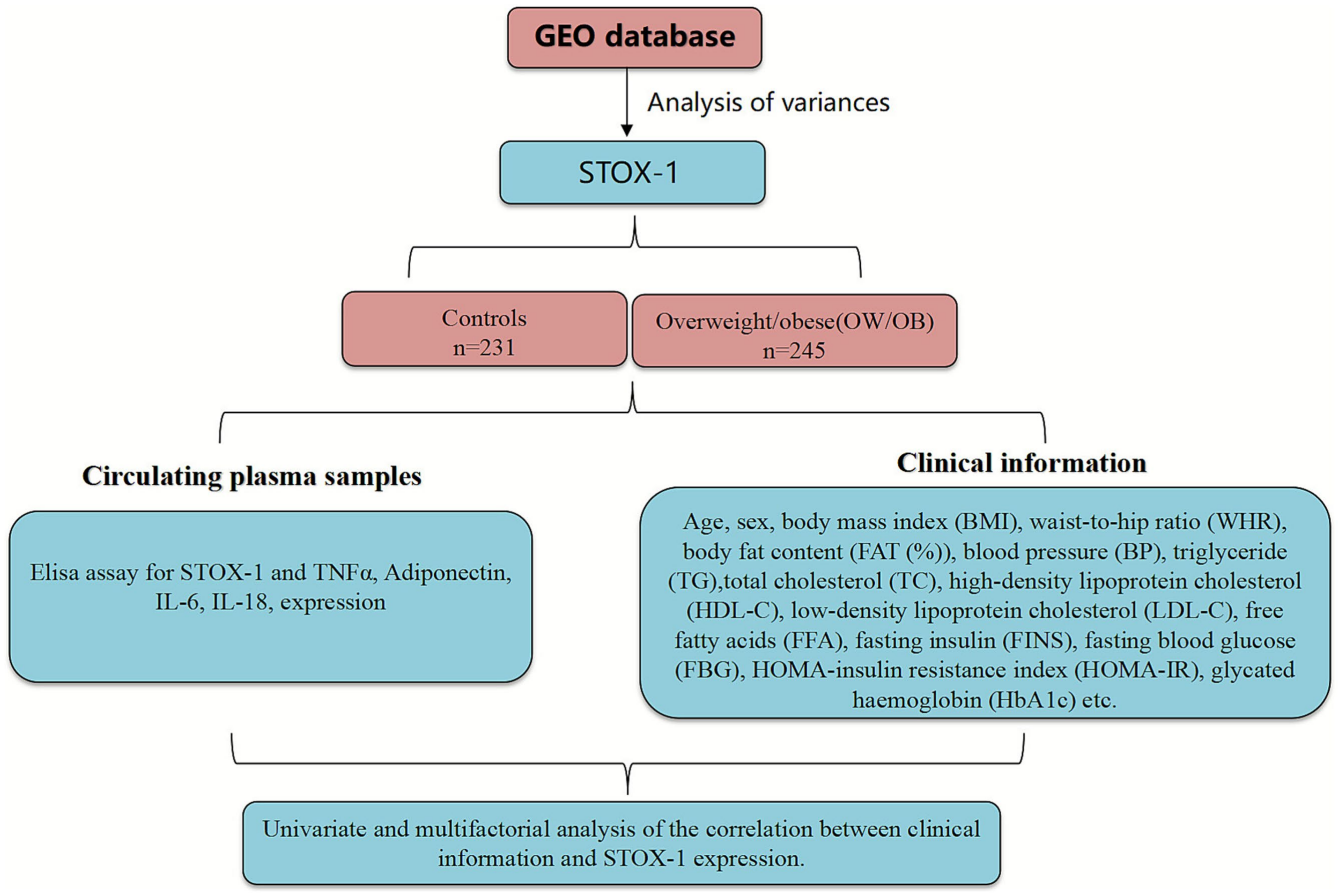
Clinical experimental design. STOX1, Storkhead box 1; OW/OB, Overweight/obese; TNF-α, Tumor necrosis factor-alpha; IL-6, Interleukin-6; IL-18, Interleukin-18; BMI, Body mass index; WHR, Waist-to-hip ratio; BP, Blood pressure; TG, Triglyceride; HDL-C, High-density lipoprotein cholesterol; LDL-C, Low-density lipoprotein cholesterol; FFA, Free fatty acids; FINS, Fasting insulin; FBG, Fasting blood glucose; HOMA-IR, HOMA-insulin resistance index; HbA1c, Glycated hemoglobins.

The study involved 476 participants aged 18 to 70, comprising 245 (OW/OB) individuals and 231 healthy controls (Figure 1), The minimum sample size was estimated based on the precision of the mean for the primary continuous variable. Using a two-sided 95% confidence level $\left(\alpha =0.05,Z_{1-\alpha /2}=1.96\right)$ and an allowable relative error of (*ε* = 0.10), the required sample size was calculated as: *n* = (Z_1-*α*/2_ *σ*/εμ)^2^. where μ and σ denote the expected mean and standard deviation, respectively. This approach has been used in similar clinical studies (18). *p* < 0.05 was considered significant in comparison to the control. According to the Chinese Diabetes Society criteria (CDS guideline 2017) for BMI (19), we divided all individuals into two groups: the normal body mass index (BMI) group (BMI < 24 kg/m^2^) and the OW/OB group (BMI ≥ 24 kg/m^2^). The participants were recruited from outpatients attending the Department of Endocrinology at Dazu’s Hospital affiliated to Chongqing Medical University, as well as from routine medical examinations, and participants had no metabolic diseases and normal liver and kidney function. Participants did not use medications that alter glucose and lipid metabolism in the past 3 months. Other metabolic disorders, including well-diagnosed diabetes, coronary heart disease (CHD), and hypertension, were listed as exclusion criteria. The study was approved by the Human Research Ethics Committee of Chongqing Medical University, following the Declaration of Helsinki (20), and registered at the Chinese Clinical Trial Registry (CCTR).

### 2.2 Clinical physical examination

All subjects had their weight and height measured, and the BMI was calculated as weight (kg)/height (m^2^). The waist circumference (WC) was measured at the narrowest indentation between the 10th rib and the iliac crest. After the patient had remained seated and at rest for 20 min, brachial blood pressure was assessed using a standard cuff and the measurement technique recommended by the American Heart Association (AHA) (21). Blood pressure (BP) was measured with a manual sphygmomanometer, and the reported value represents the mean of the second and subsequent readings. The bio-impedance method measured Body fat percentage (bia-101; RJL system, Shenzhen, China). The homeostasis model assessment of insulin resistance (HOMA-IR) was calculated using the following equations: HOMA-IR = (fasting insulin (FIns, mU/L) × fasting blood glucose (FBG, mM))/22.5 (22). After at least 8 h of overnight fasting, venous blood samples were collected from all subjects, and serum was centrifuged, separated, and stored in a − 80 °C refrigerator for subsequent experiments. All measurements were performed by trained clinical staff using standardized protocols.

### 2.3 Oral glucose tolerance test (OGTT)

OGTTs were conducted at 8:00 a.m., 10 to 12 h after fasting, with 75 grams of glucose administered to all subjects. Blood samples were collected at 0, 30, 60, and 120 min to determine the concentrations of blood glucose, insulin, and serum STOX1 (23).

### 2.4 Cytokine measurements

Serum STOX1 concentration was determined using an enzyme linked immunosorbent assay (ELISA) kit following the manufacturer’s procedure (Antibodies, UK, A310638). Serum concentrations of TNF-α (Abcam, USA, ab181421), IL-6 (Abcam, USA, ab178013), Interleukin-18 (IL-18) (Abcam, USA, ab215539), and Adiponectin (Abcam, USA, ab99968) were determined according to the manufacturer’s instructions using a highly sensitive and specific ELISA kit. These kits demonstrated no significant cross-reactivity. The intra- and intraassay coefficients of variation (CV) were <5.9 and 10% for STOX1, <2.5 and <3.1% for TNF-α, <2.1 and <2.4% for IL-6, <2.7 and <14.2% for IL-18, and <8 and <12% for Adiponectin, respectively.

### 2.5 Statistical analysis

SPSS version 26.0 (IBM Corp, Armonk, NY, USA) was used for all statistical analyses. Data are presented as mean ± standard deviation (SD) for normally distributed variables and as median [interquartile range (IQR)] for non-normally distributed variables. Normality was assessed using the Kolmogorov–Smirnov test. Variables showing non-normal distributions were log-transformed prior to inferential analyses, as appropriate.

Comparisons between the two groups were analyzed by independent Student’s t-test. The Association of STOX1 with other variables was analyzed by simple and multiple correlation coefficients. Binary logistic regression analyses were used to examine the association between serum STOX1 and obesity. The receiver operating characteristic (ROC) curve was made by SPSS 26.0 for investigating the sensitivity and specificity of STOX1 to predict obesity.

## 3 Results

### 3.1 Clinical features and circulating STOX1 and adiponectin levels in study individuals

The general clinical characteristics of the study population were shown in Table 1. The results showed that body mass index (BMI), waist-to-hip ratio (WHR), FAT (%), Diastolic blood pressure (DBP), Systolic blood pressure (SBP), triglyceride (TG) and low-density lipoprotein cholesterol (LDL-C), free fatty acid (FFA), fasting blood glucose (FBG), 2 h blood glucose after glucose overload (2 h–BG), fasting insulin (FIns), 2 h insulin after glucose overload (2 h-Ins), HOMA-insulin resistance index (HOMA-IR), the area under the curve for glucose (AUCg), the area under the curve for insulin (AUCi), HbA1c and inflammatory factors (TNF-α, IL-18, IL-6) were markedly higher in OW/OB group compared with controls, while high-density lipoprotein cholesterol (HDL-C) was lower. No significant differences were observed in age, total cholesterol (TC) between control subjects and OW/OB patients (Table 1).

**Table 1:** Main clinical features and serum STOX1 levels in the study population.

Characteristics	Overall (*n* = 476)	Overweight/obese (OW/OB)		*p*
		No (*n* = 231)	Yes (*n* = 245)	
Age (years)	51 (44–56)	51 (44–55)	51 (46–56)	0.81
Female	283	132	151	–
BMI (kg/m^2^)	25.18 (22.68–27.30)	22.64 (20.82–23.65)	27.22 (26.02–29.33)	<0.001
WHR	0.84 ± 0.07	0.82 ± 0.07	0.85 ± 0.07	<0.001
FAT (%)	29.84 ± 5.06	27.53 ± 3.95	32.01 ± 5.04	<0.001
SBP (mmHg)	110.26 ± 10.78	106.65 ± 9.54	113.78 ± 10.71	<0.001
DBP (mmHg)	72.94 ± 7.08	71.58 ± 6.97	74.22 ± 6.95	<0.001
FBG (mmol/L)	6.68 ± 1.30	6.34 ± 1.34	7.00 ± 1.18	<0.001
2 h-BG (mmol/L)	11.75 (7.00–14.20)	7.10 (6.20–13.30)	12.80 (10.50–15.15)	<0.001
FIns (mU/L)	12.34 ± 2.07	11.52 ± 2.32	13.12 ± 1.44	<0.001
2 h-Ins (mU/L)	75.15 (40.80–80.00)	39.60 (34.10–78.10)	77.30 (72.60–80.70)	<0.001
TG (mmol/L)	1.84 ± 0.52	1.60 ± 0.53	2.06 ± 0.41	<0.001
TC (mmol/L)	4.82 ± 0.71	4.78 ± 0.76	4.86 ± 0.66	0.22
HDL-C (mmol/L)	1.26 ± 0.26	1.29 ± 0.28	1.24 ± 0.23	<0.05
LDL-C (mmol/L)	2.95 ± 0.18	2.87 ± 0.19	3.02 ± 0.13	<0.001
FFA (μmol/L)	0.56 ± 0.21	0.45 ± 0.23	0.67 ± 0.12	<0.001
HOMA-IR	3.93 (2.70–4.56)	2.99 (2.21–4.42)	4.14 (3.58–4.66)	<0.001
AUCg (mmol×h/L)	29.85 (15.23–32.53)	15.15 (13.95–31.70)	30.73 (28.21–32.95)	<0.001
AUGi (mU × h/L)	133.06 (69.13–136.81)	68.43 (64.98–136.18)	134.50 (130.98–137.29)	<0.001
HbA1c (%)	7.59 ± 2.05	6.81 ± 2.03	8.33 ± 1.79	<0.001
STOX1 (ug/L)	1.02 ± 0.53	1.34 ± 0.51	0.73 ± 0.33	<0.001
Adiponectin (mg/L)	27.45(23.20–39.75)	41.50(26.10–52.90)	25.20(21.40–28.85)	<0.001
TNF-α (ng/L)	58.56 ± 13.23	50.88 ± 13.32	65.81 ± 8.07	<0.001
IL-18 (ng/L)	105.33 ± 31.64	94.51 ± 36.82	115.52 ± 21.32	<0.001
IL-6 (ng/L)	4.16 ± 1.14	3.69 ± 1.29	4.60 ± 0.73	<0.001

BMI, body mass index; WHR, waist-to-hip ratio; SBP, systolic blood pressure; DBP, diastolic blood pressure; FBG, fasting blood glucose; 2 h-BG, 2 h blood glucose after glucose overload; FIns, fasting insulin; 2 h-Ins, 2 h insulin after glucose overload; TG, triglyceride; TC, total cholesterol; HDL-C, high-density lipoprotein cholesterol; LDL-C, low-density lipoprotein cholesterol; FFA, free fatty acid; HOMA-IR: HOMA-insulin resistance index; AUCg, the area under the curve for glucose; AUGi, the area under the curve for insulin; HbA1c, glycosylated hemoglobin; STOX1, Storkhead box 1; TNF-α, tumor necrosis factor-alpha; IL-18, Interleukin-18; IL-6, Interleukin-6. Values were given as means ± SD or median (interquartile Range). Normality was assessed using the Kolmogorov–Smirnov test. Comparisons between two groups were performed using an independent (unpaired), two-tailed Student’s *t*-test.

**Figure 2:**
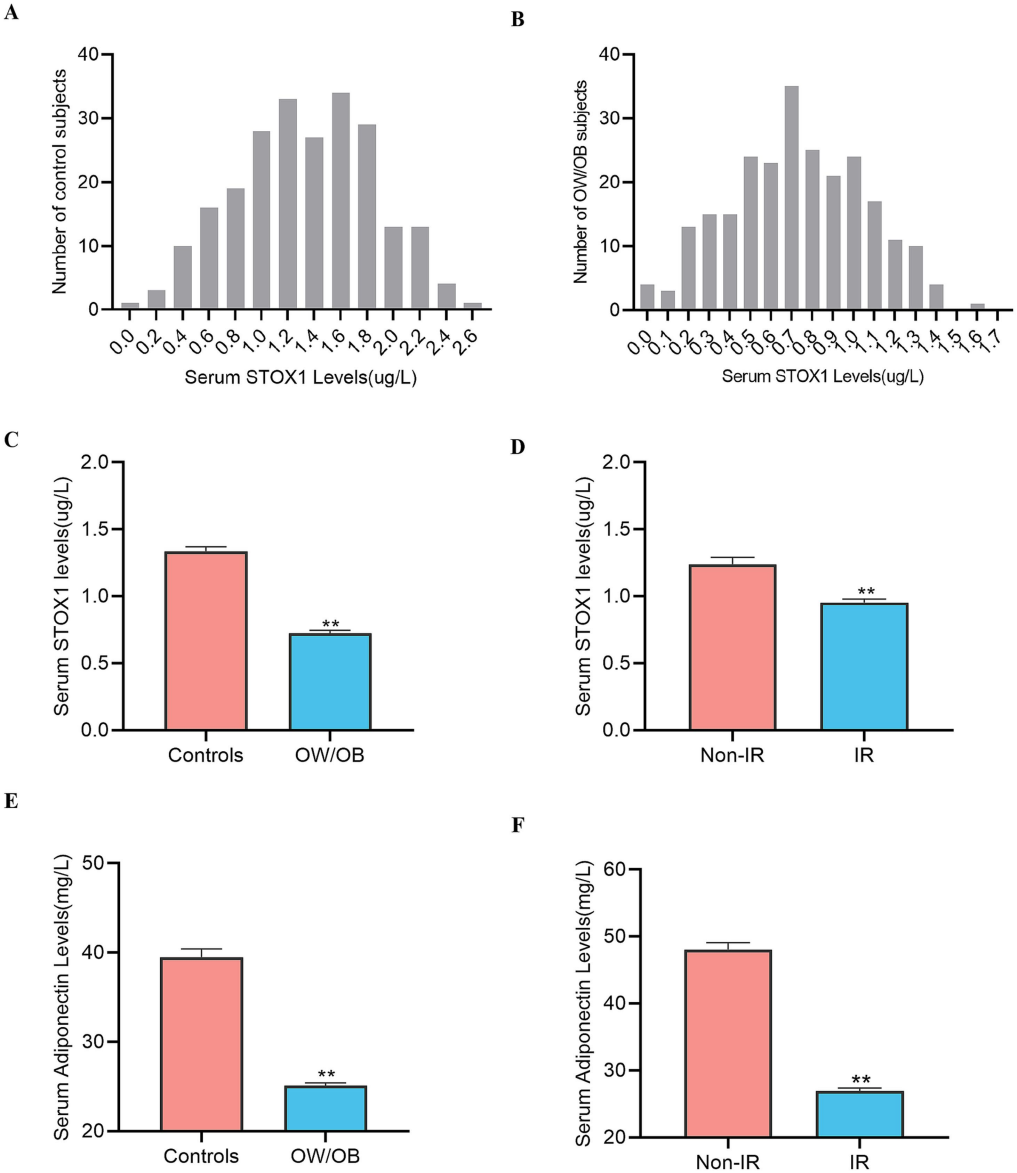
Analysis of serum STOX1 levels by different statistical approaches. **(A)** Distribution range of serum STOX1 concentration in the controls individuals. **(B)** Distribution range of serum STOX1 concentration in the OW/OB individuals. **(C)** Serum STOX1 levels in controls and OW/OB. **(D)** Serum STOX1 levels in non-insulin resistance (non-IR) and IR. **(E)** Serum adiponectin levels in controls and OW/OB. **(F)** Serum adiponectin levels in non-IR and IR. Values are shown as the mean ± SEM. **Represents significance compared to the Controls or Non-IR (**p < 0.01); Storkhead box 1 (STOX1); Overweight/Obese (OW/OB); Non-insulin resistance (Non-IR); insulin resistance (IR). Normality was assessed using the Kolmogorov–Smirnov test. Comparisons between two groups were performed using an independent (unpaired), two-tailed Student’s *t*-test.

Next, we analyzed the concentration distribution of serum STOX1 levels in two groups and found that STOX1 concentration ranged from 1.27 to 1.40 μg/L for the 95% control population (Figure 2A) and ranged from 0.69 to 0.77 μg/L for the 95% OW/OB individuals (Figure 2B). Furthermore, from OW/OB patients, serum STOX1 and Adiponectin levels were significantly decreased compared with healthy individuals (Table 1 and Figures 2C,E). In addition, based on the HOMA-IR, the population was classified into two groups: Insulin resistance (IR) group HOMA-IR ≥ 2.69 and normal group (Non-IR) HOMA-IR < 2.69. The levels of circulating STOX1 in IR individuals were notably lower than those in normal individuals [0.89 (0.59–1.24) vs. 1.18 (0.80–1.64) μg/L, *p* < 0.01] (Figure 2D), and serum Adiponectin levels were significantly lower [25.70 (22.10–29.65) vs. 51.45 (44.95–54.65) mg/L, *p* < 0:01] (Figure 2F).

### 3.2 Correlations between circulating STOX1 and other parameters as well as the occurrence of OW/OB

Then, we performed a partial correlation analysis to explore the factors that influence circulating STOX1 levels. We found that circulating STOX1 was negatively correlated with WHR, FAT (%), TG, LDL-C, FFA, FBG, 2 h-BG, FIns, 2 h-Ins, HOMA-IR, AUCg, AUCi, HbA1c, TNF-α, IL-6, and IL-18, while positively correlated with Adiponectin (Table 2 and Supplementary Figure 1). Multivariate correlation analyses revealed that FAT (%), SBP and TNF-α were independently influential factors for serum STOX1 (Table 2). The equation of STOX1 was Y_STOX1_ = 2.569–0.110 × SBP – 0.272 × TNF-α – 0.106 × FAT (%) (*R* = 0.37, *R*^2^ = 0.13). Moreover, multivariate logistic regression found that even if some variables were controlled, STOX1 is closely related to OW/OB (Table 3).

**Table 2:** The correlations analysis of variables associated with serum STOX1 levels in study population.

Variable	Simple		Multiple	
	*r*	*p*	*b*	*p*
Age (years)	0.060	0.22		
WHR	−0.118	<0.05		
FAT (%)	−0.219	<0.01	−0.106	<0.05
SBP (mmHg)	−0.186	<0.01	−0.110	<0.05
DBP (mmHg)	−0.090	0.06		
TG (mmol/L)	−0.264	<0.01		
TC (mmol/L)	−0.010	0.92		
HDL-C (mmol/L)	0.040	0.44		
LDL-C (mmol/L)	−0.254	<0.01		
FFA (μmol/L)	−0.276	<0.01		
FBG (mmol/L)	−0.168	<0.01		
2 h-BG (mmol/L)	−0.207	<0.01		
FIns (mU/L)	−0.208	<0.01		
2 h-Ins (mU/L)	−0.252	<0.01		
HOMA-IR	−0.198	<0.01		
AUCg (mmol × h/L)	−0.225	<0.01		
AUGi (mU × h/L)	−0.248	<0.01		
HbA1c (%)	−0.209	<0.01		
Adiponectin (mg/L)	0.319	<0.01		
TNF-α (ng/L)	−0.335	<0.01	−0.272	<0.01
IL-18 (ng/L)	−0.187	<0.01		
IL-6 (ng/L)	−0.236	<0.01		

In pearson correlation analysis, values included for analysis were Age, WHR, waist-to-hip ratio; SBP, systolic blood pressure; DBP, diastolic blood pressure; FBG, fasting blood glucose; 2 h-BG, 2 h blood glucose after glucose overload; FIns, fasting insulin; 2 h-Ins, 2 h insulin after glucose overload; TG, triglyceride; TC, total cholesterol; HDL-C, high-density lipoprotein cholesterol; LDL-C, low-density lipoprotein cholesterol; FFA, free fatty acid; HOMA-IR: HOMA-insulin resistance index; AUCg, the area under the curve for glucose; AUGi, the area under the curve for insulin; HbA1c, glycosylated hemoglobin; TNF-α, tumor necrosis factor-alpha; IL-18, Interleukin-18; IL-6, Interleukin-6. Multiple stepwise regression showing variables with significant independent associations with serum STOX1.

**Table 3:** Association of serum STOX1 with overweight/obese in fully adjusted models.

Model adjustment	Overweight/obese		
	OR	95%CI	*p*
Age	0.037	0.020–0.069	<0.001
Age, gender	0.037	0.020–0.068	<0.001
Age, gender, WHR	0.038	0.021–0.070	<0.001
Age, gender, WHR, FAT (%)	0.032	0.016–0.064	<0.001
Age, gender, WHR, FAT (%), BP	0.027	0.013–0.058	<0.001
Age, gender, WHR, FAT(%), BP, lipid profile	0.020	0.008–0.049	<0.001
Age, gender, WHR, FAT (%), BP, lipid profile, FBG	0.019	0.008–0.048	<0.001
Age, gender, WHR, FAT (%), BP, lipid profile, FBG, FIns	0.018	0.007–0.047	<0.001
Age, gender, WHR, FAT (%), BP, lipid profile, FBG, FIns, HOMA-IR	0.019	0.007–0.047	<0.001
Age, gender, WHR, FAT (%), BP, lipid profile, FBG, FIns, HOMA-IR, HbA1c (%)	0.019	0.007–0.047	<0.001
Age, gender, WHR, FAT (%), BP, lipid profile, FBG, FIns, HOMA-IR, HbA1c (%), Adiponectin	0.017	0.007–0.045	<0.001
Age, gender, WHR, FAT (%), BP, lipid profile, FBG, FIns, HOMA-IR, HbA1c (%), Adiponectin, inflammatory factor	0.016	0.006–0.042	<0.001

Results of binary logistic regression analysis are presented. 95% CI, confidence interval; OR, odds ratio. Data were pooled to calculate logistic regression.

**Figure 3:**
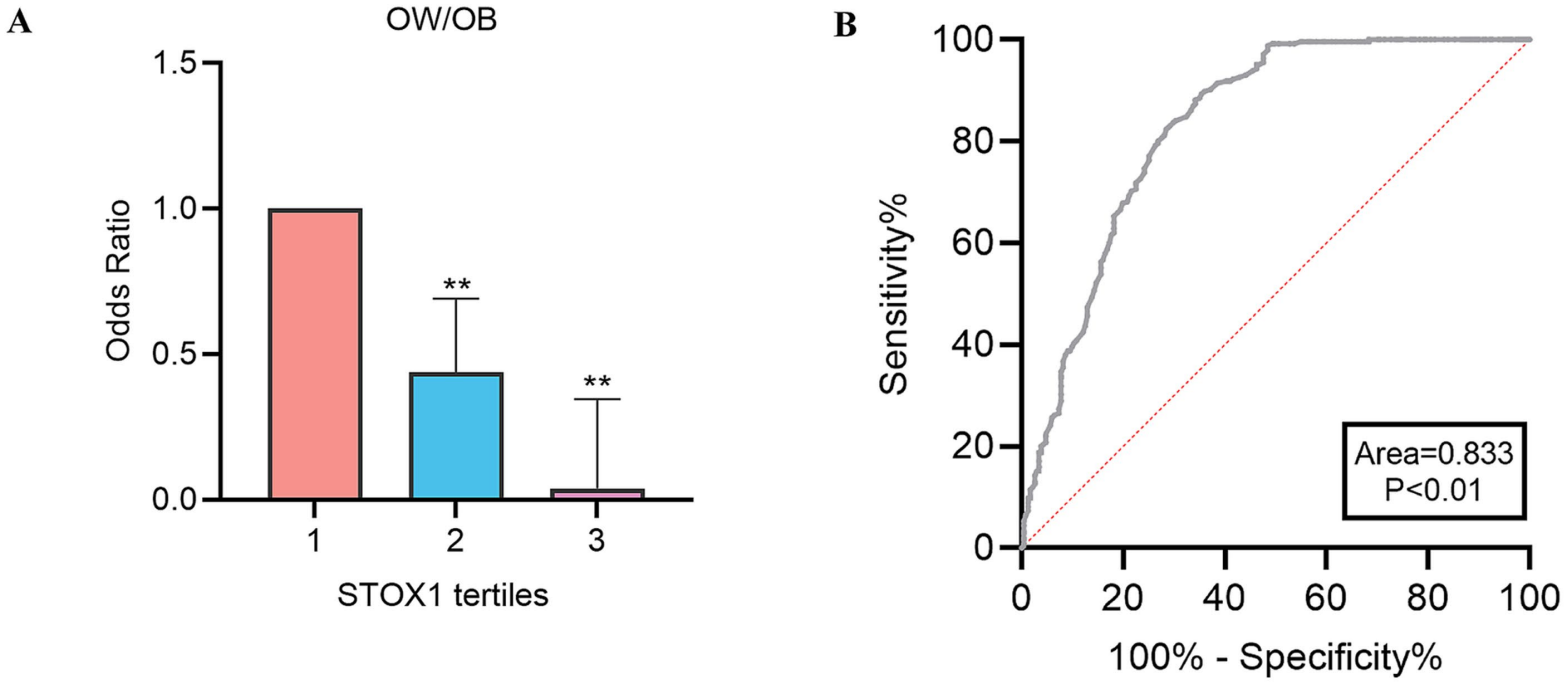
Association between serum Storkhead box 1 (STOX1) levels and obesity risk. **(A)** Odds ratios (ORs) for overweight/obesity (OW/OB) across tertiles of serum STOX1 concentration. **(B)** Receiver operating characteristic (ROC) curve analysis evaluating the predictive performance of serum STOX1 levels for OW/OB. Data are means ± SD or means ± SEM. **represents significance compared to the tertile 1 (**p < 0.01).

Next, we divided STOX1 into three tertiles (tertile 1, < 0.73 μg/L; tertile 2, 0.73–1.18 μg/L; tertile 3, >1.18 μg/L). We found that when serum STOX1 levels were in tertile 2 and 3, the odds ratios (OR) of having OW/OB were 0.437 (95% confidence interval (CI), 0.265;0.718) and 0.038 (95% CI, 0.021; 0.070) (Figure 3A and Supplementary Table 1).

Finally, the ROC curve was used to evaluate the predictive ability of STOX1 for OW/OB. The area under the ROC curve for OW/OB (AUC_OW/OB_) was 0.833 with 66% specificity and 88% sensitivity (Figure 3B). The cut-off points for serum STOX1 to predict OW/OB were 1.13 μg/L.

### 3.3 Circulating STOX1 concentration in OGTT

**Figure 4:**
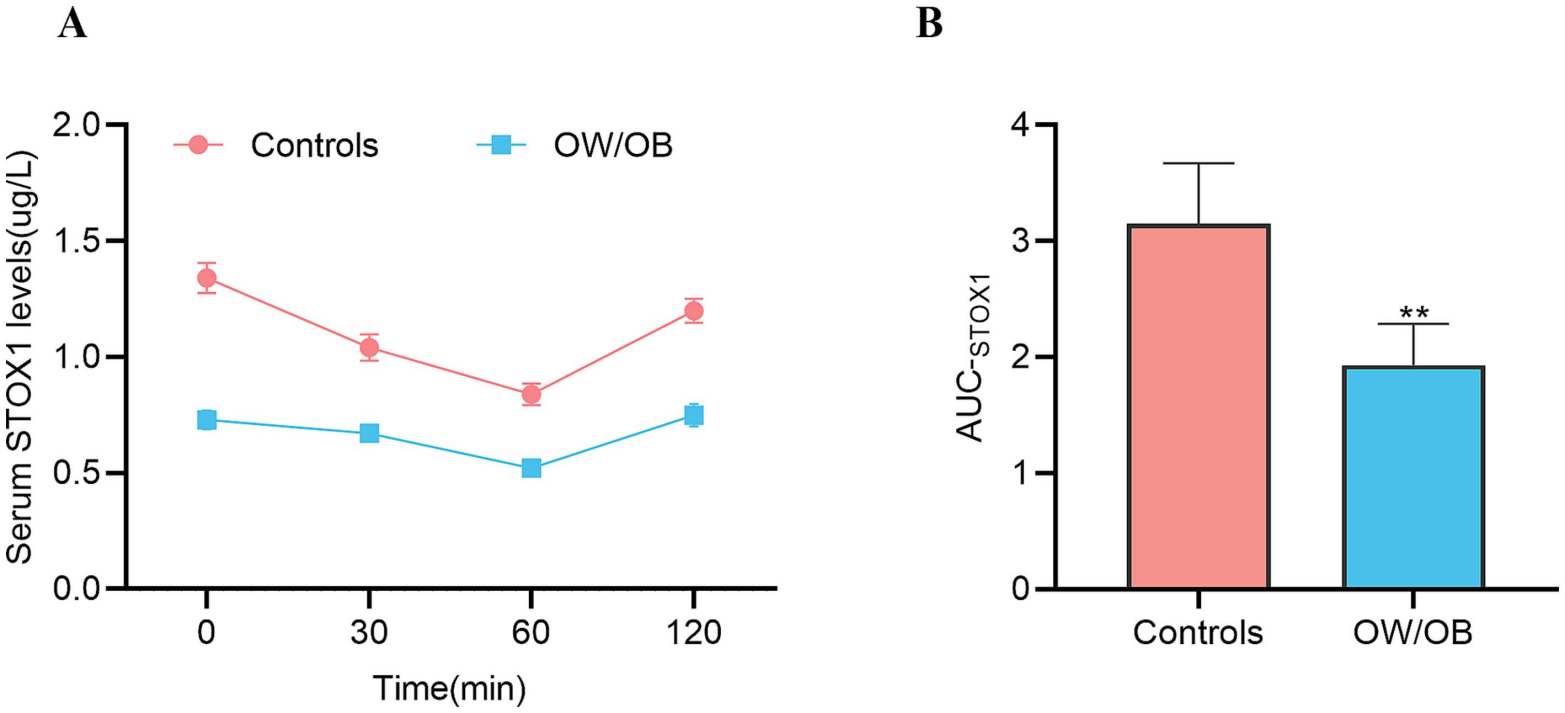
Circulating Storkhead box 1 (STOX1) concentration in Oral glucose tolerance tests (OGTT). **(A)** STOX1 concentration during OGTT. **(B)** AUCS_TOX1_ during the OGTT. Data are means ± SD or means ± SEM. **Represents significance compared to the controls. **p < 0.01. Normality was assessed using the Kolmogorov–Smirnov test. Comparisons between two groups were performed using an independent (unpaired), two-tailed Student’s *t*-test.

To explore the effects of blood glucose on circulating STOX1 levels, Oral glucose tolerance tests (OGTT) experiments were conducted in all study participants. The results showed that serum STOX1 levels change significantly during the OGTT in both normal controls and OW/OB individuals [from 1.34 ± 0.51 to 1.04 ± 0.44ug/L at 30 min, then to 0.84 ± 0.37ug/L at 60 min, and 1.20 ± 0.41ug/L at 120 min for the controls and from 0.73 ± 0.33 to 0.67 ± 0.27ug/L at 30 min, then to 0.52 ± 0.23ug/L at 60 min, and 0.75 ± 0.39ug/L at 120 min for the OW/OB (Figure 4A and Supplementary Table 2), while AUC_STOX1_ in OW/OB patients was significantly lower than that of controls (Figure 4B)]. These results indicate that hyperglycemia is associated with lower circulating STOX1 during OGTT.

## 4 Discussion

Adipokines are signaling molecules secreted by adipose tissue that play a crucial role in various physiological functions, including energy balance, metabolism, inflammation, and immune function. Adipokines play an important role in maintaining metabolic homeostasis and regulating various physiological activities and functions. Further research on adipokines and their interactions with different tissues and organs may provide valuable insights into the pathogenesis of obesity and related diseases, as well as potential therapeutic targets for these conditions. The previous study have verified that the metabolism-related gene STOX1 could effectively identify obese and thin individuals through adipose bioinformatics and machine learning, and RT-qPCR analysis also verified the downregulation of STOX1 in obese mouse models (8). Therefore, in this study, we have added clinical research, and measured the serum concentrations of STOX1 and adipokine, and Clinical features levels and assessed the serum STOX1 levels relevance in obesity. OGTT was performed to evaluate how blood glucose affects circulating STOX1 levels.

Clinical characteristics of found the FAT%, FFA, Fins, HOMA-IR, and inflammatory factors (TNF-α, IL-18, IL-6) were markedly higher in OW/OB group compared with controls, consistent with the previous findings (24), Adipose tissue is an insulin-sensitive endocrine organ that plays a crucial role in glucose and lipid metabolism by buffering daily fluctuations of fatty acids during the postprandial period and by secreting adipokines (25). The mainly role of insulin in adipose tissue is to suppress lipolysis and to promote glucose uptake and lipogenesis, thereby regulating secretion of FFAs into the bloodstream (26). In our study, we observed increases in both circulating insulin and FFA levels (Table 1). This may be attributed to the resistance of adipose tissue to the anti-esterification effects of insulin in cases of obesity and insulin resistance. This resistance leads to an inability to effectively inhibit the release of FFA, resulting in a continuous increase in circulating FFA levels (3). Adipose tissue is essential for maintaining the body’s metabolic balance. In individuals with long-term obesity, an increase in adipose tissue mass occurs due to the accumulation of triglycerides in fat cells, resulting in the enlargement of these cells (27). Multiple mouse models of obesity have shown that adipose tissue contributes to whole-body insulin resistance, as indicated by insufficient oxygen supply to adipocytes and increased oxygen demand from adipocytes leading to hypoxia (28, 29), which stimulates adipose tissue fibrogenesis and macrophage chemotaxis (30, 31). Increasing the number and relative proportion of adipose tissue proinflammatory immune cells (macrophages and T cells) and the expression of the genes that encode proinflammatory proteins, lipolysis of adipose tissue and release of FFAs into the circulation but decreasing adipose tissue production and secretion of adiponectin (32–34). Similar results were also observed in the subcutaneous adipose tissue of obese individuals our findings are consistent with this (35, 36). No significant differences in age and total cholesterol were observed between control subjects and OW/OB patients. This suggests that age and total cholesterol may not be the main factors causing obesity.

Serum analysis indicated that circulating levels of STOX1 and adiponectin were significantly lower in obese individuals compared to the control group. Notably, similar findings were observed in insulin-resistant patients as well as in individuals with normal insulin sensitivity. Plasma adiponectin levels are often inversely related to body fat percentage and directly related to insulin sensitivity (33), and can be considered a biomarker of adipose tissue health (37). In rodents, adiponectin exhibits anti-inflammatory, anti-fibrotic, and insulin-sensitizing effects while promoting the survival and regeneration of pancreatic *β* cells (38). The therapeutic effects of adiponectin are likely mediated by increased ceramidase activity and decreased intracellular ceramide levels, which occur when adiponectin binds to its cell surface receptors 1 and 2 (39). Therefore, we speculate that STOX1 may be involved in adipose cells inflammation and insulin sensitization processes, thereby affecting adipocyte inflammation or insulin resistance. Chronic, low-grade inflammation, characterized by an increase in pro-inflammatory immune cells and the expression of genes encoding pro-inflammatory proteins, is observed in people with obesity. Under normal circumstances, the increase in adipose tissue gene expression does not always lead to higher plasma concentrations of the encoded proteins, and many of these cytokines function locally. A recent study evaluated plasma cytokine levels every hour for 24 h in individuals with obesity. It compared those who were insulin-sensitive to those who were insulin-resistant. The study found no differences in the 24-h plasma concentration area-under-the-curve measurements for a range of cytokines, except for plasminogen activator-1 (PAI-1) (36). In contrast, we observed that serum TNF-α, IL-18, and IL-6 levels were significantly higher in OW/OB participants than in normal-weight controls. These pro-inflammatory cytokines were negatively correlated with STOX1, but—unexpectedly—were positively correlated with adiponectin, a finding that may reflect differences in participant characteristics and metabolic status. Under physiological conditions, adiponectin is typically inversely associated with systemic inflammation; however, this relationship can be altered in settings of chronic inflammation and metabolic dysfunction, such as advanced obesity, severe insulin resistance, and type 2 diabetes. Consistent with our observation of heightened inflammation in OW/OB individuals, Gart et al. (40). reported elevated circulating TNF-α, IL-6, and IL-18 in obesity and suggested that these markers are closely linked to increased fat mass, particularly visceral adiposity, and adipose-tissue remodeling; importantly, reductions in fat mass (e.g., after bariatric/metabolic surgery) may attenuate this inflammatory profile. Moreover, obesity-associated oxidative stress can promote LDL oxidation to ox-LDL, thereby amplifying inflammatory signaling through endothelial activation and subsequent immune-cell recruitment and infiltration.

STOX1 is an angle encryption factor that shares structures and functional comparisons with disguise transfer factor (9). STOX1 encodes six isoforms, among which STOX1A and STOX1B are the most extensively studied. Research on STOX1 has primarily focused on its involvement in various biological processes, including cell cycle regulation (14), early embryonic development (41), and the modulation of oxidative stress (15). Notably, the most intensively investigated aspect of STOX1 pertains to its role in preeclampsia and placental function (42). However, relatively few studies have explored its direct metabolic associations with adiponectin, leptin, or inflammatory cytokines. Preeclampsia is characterized by placental inflammation, oxidative stress, and alterations in maternal metabolism, including changes in adiponectin and leptin levels. In a mouse model overexpressing STOX1, genes associated with inflammation and cellular stress were significantly upregulated in endothelial cells (ECs) (43). Systems biology analyses further identified interleukin-6 (IL-6) as a central regulatory node, suggesting that STOX1 may contribute to the pathogenesis of preeclampsia by modulating inflammatory pathways (44). Collectively, these observations imply that STOX1 has an overall anti-inflammatory effect that is suppressed in obese individuals. Our data also demonstrates that circulating STOX1 levels are significantly decreased in overweight and obese (OW/OB) patients and show a negative correlation with HOMA-IR levels, indicating a potential link between STOX1 and metabolic dysfunction. In summary, although the precise biological function of STOX1 remains to be fully elucidated, current evidence suggests that its expression and secretion are upregulated in dysfunctional adipose tissue during obesity. STOX1 may play a modulatory role in obesity-associated inflammation and contribute to the development of obesity-linked metabolic adaptations. Further preclinical studies are warranted to clarify these mechanistic pathways.

Partial correlation analysis revealed that circulating STOX1 levels were negatively associated with metabolic and inflammation-related markers and positively associated with adiponectin. Adiponectin is well-established as an insulin-sensitizing adipokine. In individuals with type 2 diabetes and obesity, circulating adiponectin levels are typically reduced but increase following improvements in insulin sensitivity (45–47). Consistent with previous findings, we observed decreased circulating adiponectin concentrations in overweight and obese (OW/OB) subjects. Furthermore, multivariate correlation analysis identified systolic blood pressure (SBP) as an independent factor influencing serum STOX1 levels. These findings further support the association between STOX1 and insulin resistance (IR), suggesting that STOX1 may function as an independent regulatory node in the modulation of insulin sensitivity. To further investigate whether circulating STOX1 levels are regulated by blood glucose, we conducted an oral glucose tolerance test (OGTT). Following oral glucose administration, serum STOX1 levels were significantly lower in overweight and obese (OW/OB) patients compared to controls. Moreover, the total area under the curve (AUC) for STOX1 was markedly reduced in OW/OB patients, indicating that hyperglycemia may inhibit STOX1 secretion and release. Previous studies have shown that elevated glucose levels significantly increase the secretion of CXC chemokines—such as CXCL1 and CXCL8 (also known as IL-8)—in placental trophoblast cells, thereby enhancing the inflammatory response (48). Lekva et al. (49) recently reported that women with gestational diabetes mellitus (GDM) have a higher risk of neonatal infection due to placental abnormalities, In particular, a high-glucose environment was shown to activate the toll-like receptor 4 (TLR4)/myeloid differentiation primary response 88 (MyD88)/NF-κB signaling pathway in GDM placentas, leading to increased secretion of CXCL8/IL-8. The upregulation of inflammatory mediators such as IL-8 may initiate inflammatory cascades within the placenta through specific gene transcription or translational mechanisms. By analogy, elevated circulating glucose levels may enhance IL-8 secretion from adipocytes through similar signaling pathways, potentially contributing to the reduction in circulating STOX1 levels. Further cellular and animal studies are warranted to elucidate the mechanistic relationship between STOX1, metabolic dysregulation, and obesity.

This study evaluated the association between STOX1 protein expression and obesity or insulin resistance (IR), and its potential links to glycemic dysregulation and inflammatory signaling. We observed that STOX1 levels were reduced in adipose tissue from obese/IR individuals, accompanied by elevated pro-inflammatory mediators (e.g., IL-6). Moreover, in obese participants, STOX1 expression in adipocytes from peripheral adipose depots was inversely correlated with pro-inflammatory cytokines, including IL-6 and IL-8, supporting the hypothesis that STOX1 may contribute to restraining cytokine release and thereby mitigating adipose-tissue inflammation. To our knowledge, this is the first report to describe this specific STOX1–inflammation relationship in the context of metabolic disease, and ongoing studies are aimed at elucidating the underlying mechanisms.

However, several limitations should be acknowledged in this study: (1) Although the overall sample size was relatively large, the number of patients within each subgroup remained limited—a common constraint in cohort-based analyses. (2) The study was conducted exclusively in a Chinese population; therefore, caution is warranted when generalizing these findings to other ethnic groups. (3) Owing to its cross-sectional design, the study cannot establish a causal relationship between STOX1 expression and obesity.

## 5 Conclusion

In summary, our findings highlight a potential link between STOX1 expression and metabolic dysregulation in obesity and insulin resistance (IR). Circulating STOX1 levels were reduced in overweight and obese individuals and showed significant correlations with inflammatory markers, adiponectin, and indicators of insulin sensitivity. Notably, STOX1 expression in adipose tissue was negatively associated with pro-inflammatory cytokines such as IL-6 and IL-8, suggesting a role in modulating adipose tissue inflammation. Additionally, oral glucose tolerance testing indicated that hyperglycemia may inhibit STOX1 secretion, further implicating STOX1 in glucose-responsive inflammatory signaling. These novel observations suggest that STOX1 may serve as a regulatory factor in obesity-related inflammation and metabolic disorders. However, further mechanistic studies are needed to elucidate the causal pathways and therapeutic potential of STOX1 in metabolic disease article types.

## Data Availability

The original contributions presented in the study are included in the article/Supplementary material, further inquiries can be directed to the corresponding authors.
