# Supplementary material for: Reduced circulating STOX1 is associated with inflammatory cytokines and insulin resistance in obese individuals: a cross-sectional study
Source: Front Nutr. 2026 Jan 22;13:1734606. doi: 10.3389/fnut.2026.1734606 (PMC12872547; doi:10.3389/fnut.2026.1734606)
Supplement: Supplementary file 1 [file Table_1.DOCX]

Supplementary Material

# Supplementary Data

Supplementary Material should be uploaded separately on submission. Please include any supplementary data, figures and/or tables.

Supplementary material is not typeset so please ensure that all information is clearly presented, the appropriate caption is included in the file and not in the manuscript, and that the style conforms to the rest of the article.

# Supplementary Figures and Tables

For more information on Supplementary Material and for details on the different file types accepted, please see [here](https://www.frontiersin.org/guidelines/author-guidelines#supplementary-material).

## Supplementary Figures

**Supplementary Figure1**


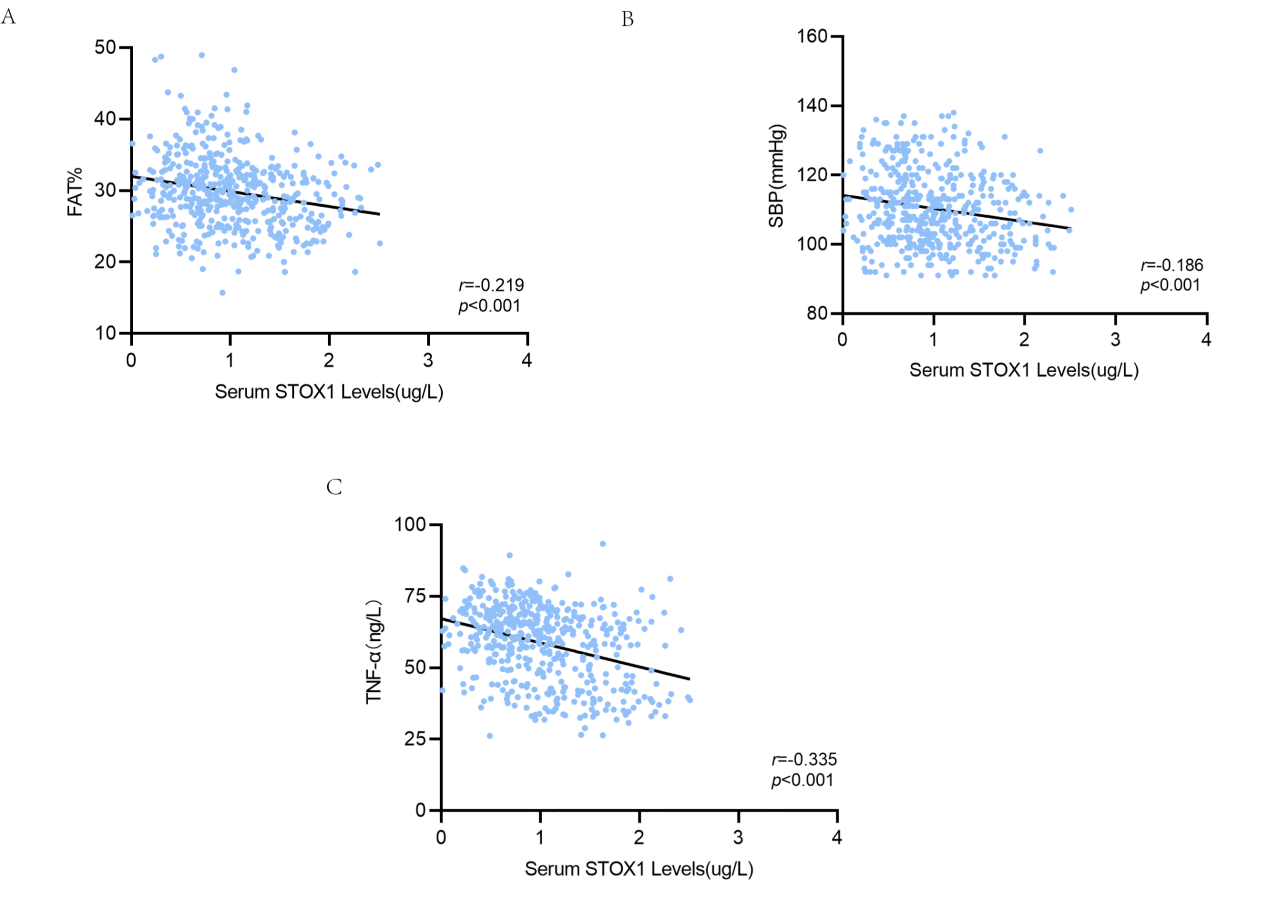


**Supplementary Figure1** Serum levels of STOX1 reduced with obesity in relation to inflammation of adipose tissue. (A) Serum STOX1 correlates with FAT%. (B) Serum STOX1 correlates with SBP. (C) Serum STOX1 correlates with TNF-α.

## Supplementary Tables

| **group** | **STOX1** | **Univariable** | | |
| --- | --- | --- | --- | --- |
|  |  | **OR** | **95%CI** | **P** |
| OW/OB | STOX1,categorical(Tertile 1 as reference) | 1 | - | - |
|  | Tertile 2 | 0.437 | 0.265-0.718 | <0.01 |
|  | Tertile 3 | 0.038 | 0.021-0.070 | <0.01 |

**Table1 Association of different concentrations of serum STOX1 level in OW/OB subjects**

OR:odd ratio,95%CI:95%confidence interval.

**Table2 Serum STOX1 levels in Controls and OW/OB subjects from minutes 0 to 120min during OGTT**

| **STOX1(ug/L)** | **0min** | **30min** | **60min** | **120min** |
| --- | --- | --- | --- | --- |
| Controls(n=231) | 1.34±0.51 | 1.04±0.44* | 0.84±0.37** | 1.20±0.41** |
| OW/OB(n=245) | 0.73±0.33 | 0.67±0.27** | 0.52±0.23** | 0.75±0.39** |

Values were given as means ± SD. *p<0.05,**p < 0.01 compared with 0min.
